# A Coupled Resonator Optical Waveguide-Based Refractive Index Sensor Employing Sagnac Loop Reflectors

**DOI:** 10.3390/s26051448

**Published:** 2026-02-26

**Authors:** Muhammad A. Butt, Bartosz Janaszek

**Affiliations:** Institute of Microelectronics and Optoelectronics, Warsaw University of Technology, Koszykowa 75, 00-662 Warsaw, Poland; bartosz.janaszek@pw.edu.pl

**Keywords:** Sagnac loop reflector, refractive index sensor, subwavelength grating, silicon-on-insulator, coupled resonator optical waveguide

## Abstract

This work presents a silicon-on-insulator (SOI) refractive index sensor based on a coupled resonator optical waveguide (CROW) architecture employing two inversely coupled Sagnac loop reflectors (SLRs) connected through a self-coupled feedback waveguide. The structure exploits bidirectional propagation and discrete–continuum interference to produce sharp Fano-type asymmetric resonances with steep spectral slopes, enabling enhanced wavelength sensitivity. Numerical analysis demonstrates that tuning the loop radius, directional-coupler length, coupling gap, and feedback-path length provides precise control over free spectral range (FSR), resonance asymmetry, and spectral sharpness. The sensor exhibits consistent and monotonic resonance shifts for refractive index variations from 1.33 to 1.36, with sensitivities ranging from 106 to 120 nm/RIU for the ridge feedback configuration. Sensitivity is further improved by introducing a subwavelength grating (SWG) segment into the feedback waveguide, which enhances evanescent-field interaction and increases the overlap factor without compromising compactness or Fano asymmetry. The SWG-assisted design attains sensitivities of 185.8–212.2 nm/RIU, nearly doubling sensitivity. The proposed coupled-SLR CROW provides a compact footprint, high-Q resonances, and flexible spectral engineering through accessible geometric parameters. These characteristics highlight the potential of the coupled-SLR and SWG-enhanced CROW as a promising platform for high-resolution, photonic refractive index sensing applications on SOI.

## 1. Introduction

Silicon (Si) photonics has emerged as a leading platform for on-chip refractive index sensing due to its CMOS compatibility, high index contrast, and ability to support compact interferometric and resonant structures with strong light–matter interaction [1,2,3,4]. Among the various integrated sensing approaches, resonator-based devices such as microring resonators [5,6], Sagnac interferometers [7,8,9], and cascaded resonant networks [10,11] have received significant attention owing to their high spectral resolution and suitability for label-free biochemical detection. However, many of these architectures suffer from sensitivity limitations, fabrication-induced phase errors, or limited tunability, which can limit their performance in practical applications that require high resolution, robustness, and compactness. As sensing requirements continue to advance, there is a growing need for architectures that combine structural simplicity with enhanced field confinement and improved control over spectral response [12].

Coupled resonator optical waveguides (CROWs) have recently attracted interest as promising candidates for high-performance refractive index sensing, leveraging multi-cavity interference to achieve sharp resonances and flexible spectral engineering within a small footprint [13]. When properly configured, CROW structures can exhibit steep transmission slopes and strong phase sensitivity, attributes that are particularly advantageous for wavelength-interrogated sensors [14]. Integrating Sagnac loop reflectors (SLRs) into CROW networks further expands their capabilities by enabling bidirectional propagation and discrete–continuum interference, giving rise to Fano-type resonances that enhance detection accuracy [15,16]. These features motivate the exploration of coupled-SLR-based CROW topologies to achieve robust, highly sensitive, and fabrication-tolerant refractive index sensors on the SOI platform [17].

An important motivation for adopting the coupled-SLR with a feedback–loop configuration for refractive index sensing lies in its structural simplicity and potential resilience to fabrication-induced perturbations [18]. Because the resonant cavity is formed through a single, continuous waveguide rather than several fully isolated resonators, dimensional variations in either the SLR section or in the feedback path are expected to primarily induce a global spectral shift rather than significantly altering the resonance lineshape. Such behavior can help preserve key spectral characteristics, including the Fano-type asymmetry, under typical silicon-on-insulator (SOI) fabrication conditions. This feature is advantageous for sensing applications, as it may reduce the need for post-fabrication adjustments and improve consistency across devices. By contrast, multi-resonator platforms can be more sensitive to relative phase errors, which can distort the spectral response and reduce sensing accuracy [19]. Furthermore, the high refractive index contrast (Δn) of the SOI platform facilitates strong optical confinement and compact loop dimensions, while also enabling enhanced evanescent-field interaction through narrow or refractive index-engineered waveguides to increase the overlap factor (Γ) and thereby enhance the wavelength sensitivity [20,21]. Taken together, the compact footprint, tunable lineshape characteristics, and strong tolerance to fabrication variations make this coupled-SLR and feedback–loop architecture a promising candidate for refractive index sensing, especially in wavelength-interrogated operation.

In this paper, we propose a compact sensing platform composed of two inversely coupled Sagnac loop reflectors linked via a feedback waveguide, and we analyze its spectral behavior using the finite element method (FEM) in COMSOL Multiphysics 6.4. We identify the mechanisms governing its Fano-type resonance and quantify how key structural parameters influence the free spectral range, lineshape asymmetry, and extinction characteristics. We further demonstrate that incorporating a subwavelength grating (SWG) feedback section markedly enhances the refractive index sensitivity. Together, these results establish a tunable and fabrication-tolerant architecture for high-performance integrated refractive index sensing.

## 2. Design and Numerical Analysis

**Figure 1:**
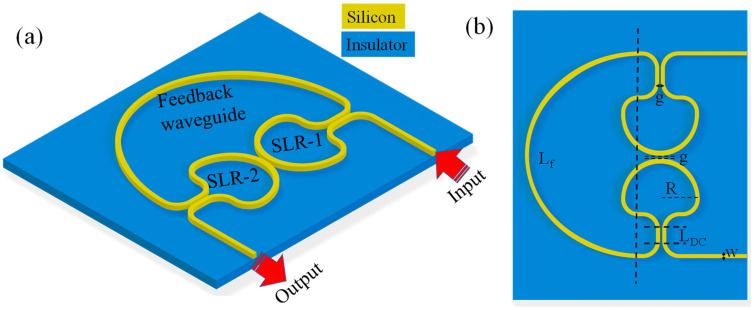
(**a**) 3D representation and (**b**) 2D representation of CROW-based sensor employing SLRs.

The sensing device is designed on the SOI platform and is based on two inversely coupled Sagnac loop reflectors (denoted as SLR-1 and SLR-2) connected through a self-coupled feedback waveguide (Figure 1a,b). Each SLR consists of a directional coupler (DC) and a waveguide loop that supports counter-propagating waves. When light enters an SLR, it splits into clockwise and counterclockwise components, which accumulate phase in the loop and then recombine at the same coupler. Their recombination produces wavelength-dependent constructive or destructive interference. By placing two such SLRs in proximity and coupling them through a central DC, the structure creates coherent mixing between the optical fields of the two loops. The resonance itself follows:

(1) $$m\lambda =n_{eff}L_{rt},$$

where m is the mode number, λ is the resonant wavelength, and L_rt_ is the effective round-trip length including contributions from both SLR loops and the feedback path. Because the cavity behaves as a standing-wave resonator, its free spectral range (FSR) is determined by the following:

(2) $$FSR\approx \frac{\lambda^{2}}{n_{g}L_{rt}},$$

where n_g_ is the group index.

In the sensing operation, the SLR loops and the feedback waveguide are exposed to the external analyte. A change in the analyte refractive index modifies the modal effective index (n_eff_) through evanescent-field interaction, and thus changes the total round-trip phase accumulated in the coupled cavity. This results in a spectral shift in the resonance, which underlies refractive index sensing. The wavelength sensitivity is expressed as

(3) $$S_{\lambda }=\frac{∆\lambda }{∆n};$$

where Δλ and Δn denote the change in resonance wavelength and the change in ambient refractive index, respectively. Although a Fano resonance exhibits an asymmetric lineshape due to interference between a discrete state and a continuum, the quality factor (Q-factor) is still defined using the standard resonance width relation. The Q-factor is expressed as [22]

(4) $$Q\text{-}factor=\frac{\lambda_{res}}{FWHM};$$

where λ_res_ is the resonance wavelength, and FWHM is the full width at half maximum of the Fano resonance. It is noteworthy that Fano resonances exhibit intrinsically asymmetric line shapes arising from interference between discrete and continuum modes, and, thus, the conventional symmetric Lorentzian FWHM is not directly applicable. Therefore, following the approach demonstrated in [22], we define the FWHM as the absolute spectral separation between the dip/resonance $\lambda_{d}$ and the peak $\lambda_{p}$ of the given resonance, i.e., $FWHM=\;\left|\lambda_{d}-\lambda_{p}\right|$, which provides a robust and physically meaningful measure of the effective resonance width for strongly asymmetric profiles.

The architecture presented in this work is particularly advantageous for refractive index sensing because it combines high spectral sharpness with low loss. Unlike conventional microring resonators, where the standard bus–ring coupling geometry excites a single propagation direction predominantly, the SLR structure inherently generates counter-propagating waves and therefore supports richer bidirectional interference. When two SLRs are coupled, the resulting interference produces strong asymmetric resonances with very steep spectral slopes. These sharp Fano features can significantly enhance practical sensitivity, since even a small resonance shift can produce a large change in transmission. Furthermore, the feedback waveguide introduces an additional phase parameter that enables flexible resonance tuning without the need for complicated multi-stage structures. Compared with other higher-order filtering architectures, such as cascaded microrings [5,23,24], ring-assisted Mach–Zehnder interferometers [25], or multi SLR chains [26], this structure achieves comparable spectral control with a significantly smaller footprint and fewer constituent resonators. This reduction in complexity not only simplifies fabrication but also enhances sensing reliability by reducing phase-matching requirements and minimizing cumulative optical loss.

To ensure consistent evaluation of device behavior across variations in loop radius, coupling length, and feedback-path configuration, the geometrical parameters of the coupled-SLR CROW sensor are summarized in Table 1. These parameters define the fundamental physical layout of the two SLRs, the inter-SLR coupling region, and the feedback waveguide. By systematically adjusting only one parameter at a time while keeping the others fixed within the ranges listed, the study isolates the individual influence of each structural component on resonance asymmetry, free spectral range, and overall spectral performance. This parameterization provides the baseline for all numerical simulations and enables a clear interpretation of the device’s spectral and sensing characteristics.

All simulations in this work were carried out using the COMSOL Multiphysics^®^ software package. The device was modeled using a two-dimensional (2D) formulation appropriate for planar SOI photonic structures. The electromagnetic response was solved using the Electromagnetic Waves, Frequency Domain interface, which implements the finite element method (FEM) to solve Maxwell’s equations in the steady-state frequency regime. To enable efficient simulation, the three-dimensional SOI waveguide was reduced to an equivalent 2D model using a standard effective-index approximation. The modal effective index, obtained from a preliminary eigenmode analysis of the vertical waveguide stack, was assigned as the core refractive index in the planar geometry. This approach accurately captures in-plane propagation and resonance behavior while significantly reducing computational cost compared to full three-dimensional simulations. A triangular finite-element mesh with a minimum element size of 50 nm was employed to accurately resolve subwavelength features and coupling regions. Scattering boundary conditions were applied at the outer domain to prevent nonphysical reflections and to emulate open boundaries. A wavelength sweep was performed across the C-band (1530 nm to 1565 nm) to evaluate the transmission characteristics and extract the resonance behavior of the structure. The Si waveguide core and SiO_2_ substrate were assigned refractive index values obtained directly from the COMSOL material library. At the operating wavelength around 1550 nm, Si and SiO_2_ exhibit refractive indices of approximately 3.48 and 1.44, respectively, providing the high index contrast required for strong optical confinement in the SOI platform.

## 3. Device Optimization

**Figure 2:**
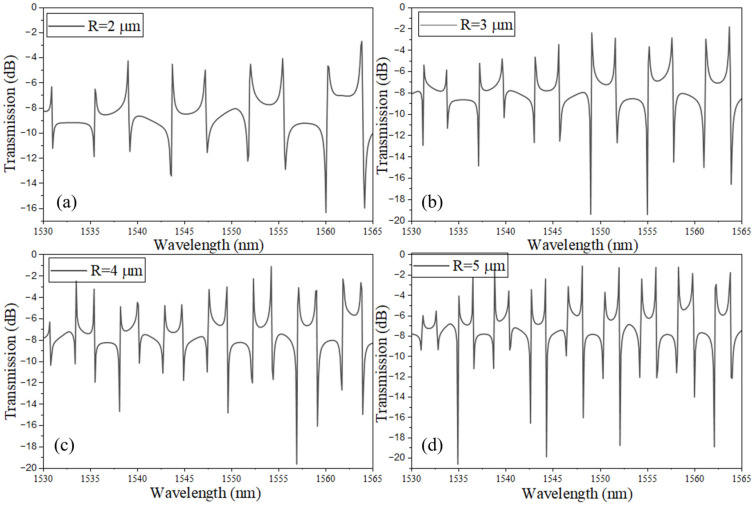
Transmission spectrum of the coupled-SLRs: (**a**) R = 2 µm, L_f_ = 46.32 µm; (**b**) R = 3 µm, L_f_ = 62.02 µm; (**c**) R = 4 µm, L_f_ = 77.72 µm; (**d**) R = 5 µm, L_f_ = 93.42 µm. Note: L_DC_ = 5 µm; g = 150 nm.

Figure 2a–d presents the simulated transmission spectra of the coupled-SLR CROW structure for different SLR radii (R), while maintaining a fixed directional-coupler length L_DC_ = 5 μm and a uniform coupling gap of g = 150 nm. Four cases are examined, corresponding to R = 2, 3, 4, and 5 μm, with the feedback-waveguide length L_f_ appropriately adjusted in each configuration to ensure efficient phase interaction within the composite cavity. As the loop radius increases, the overall round-trip optical length (L_rt_) of the device, which includes contributions from both SLR loops, the feedback path, and the directional couplers, also increases. The longer round-trip path leads to a systematic reduction in the free spectral range (FSR), resulting in a denser distribution of spectral resonances within the wavelength window. In the simulated structures, the FSR decreases from approximately 4.3 nm to 2.1 nm as R increases from 2 µm to 5 µm. The corresponding analytical predictions range from 4.67 nm to 2.14 nm. These results indicate percentage deviations of about 7.9% at R = 2 μm and 1.9% at R = 5 μm between the numerical and analytical calculations.

In all four cases, the device preserves a high-contrast Fano-type lineshape in the vicinity of each resonance. This asymmetric spectral response arises from the interference between discrete resonance pathways supported by the Sagnac loops and the quasi-broadband pathway enabled through the central coupling section and the feedback waveguide. Because L_DC_ and g are kept constant for all configurations, the coupling strength between waveguides remains nearly identical, isolating the effect of increased loop perimeter as the dominant mechanism governing changes in spectral characteristics. The observed increase in resonance density with larger R, therefore, directly reflects the longer accumulated phase per round-trip rather than variations in coupling efficiency.

Furthermore, the persistence of the Fano asymmetry across all loop radii highlights the structural robustness of the coupled-SLR configuration. Even as the cavity size increases, constructive and destructive interference among counter-propagating fields within each SLR, together with feedback-mediated mixing between the loops, yield spectral features that are similarly sharp. These steep resonance slopes are advantageous for refractive index sensing, as they enhance wavelength-shift detectability and enable high-resolution measurements.

**Figure 3:**
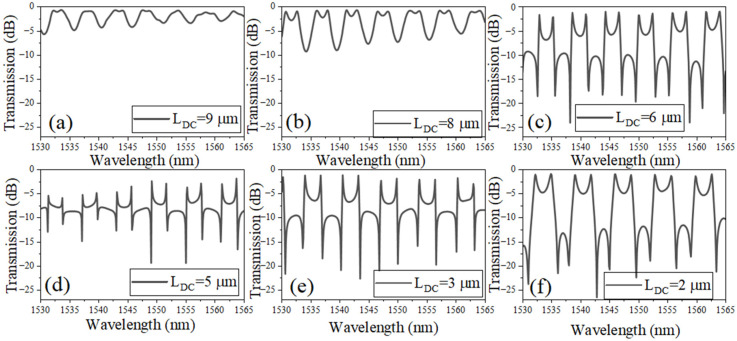
Transmission spectrum of the coupled-SLRs: (**a**) L_DC_ = 9 µm, L_f_ = 74.6 µm; (**b**) L_DC_ = 8 µm, L_f_ = 71.4 µm; (**c**) L_DC_ = 6 µm, L_f_ = 65.2 µm; (**d**) L_DC_ = 5 µm, L_f_ = 62 µm; (**e**) L_DC_ = 3 µm, L_f_ = 55.74 µm; (**f**) L_DC_ = 2 µm, L_f_ = 52.6 µm. Note: R = 3 µm; g = 150 nm.

Figure 3a–f shows the simulated transmission spectra of the coupled-SLR CROW structure for different directional-coupler lengths (L_DC_), while keeping the loop radius fixed at R = 3 μm and the coupling gap at g = 150 nm. Six values of L_DC_ are investigated: 9, 8, 6, 5, 3, and 2 μm, with corresponding feedback-waveguide lengths L_f_ adjusted to maintain phase matching within the composite cavity. A clear evolution of the spectral response is observed as L_DC_ is varied. When L_DC_ is relatively long, as in Figure 3a, the resonance profile becomes more symmetric, and the characteristic Fano asymmetry is significantly diminished. This indicates that the extended coupling region suppresses the phase imbalance between the discrete resonant path and the broadband background pathway. As L_DC_ decreases, the interference between these pathways strengthens, leading to increasingly asymmetric Fano resonances. The most pronounced Fano lineshape appears when L_DC_ = 2 μm to 6 μm, as seen in Figure 3c–f, where the transmission exhibits sharp asymmetric dips with steep slopes.

Throughout Figure 3, the free spectral range (FSR) remains nearly unchanged, since the total round-trip length is dominated by the fixed loop perimeter and the feedback-waveguide section, while variations in L_DC_ contribute only marginally to L_rt_. Thus, the primary effect of changing L_DC_ is not resonance-spacing modification but lineshape control. These results demonstrate that L_DC_ acts as an effective tuning parameter for engineering spectral asymmetry and resonance sharpness. Shorter coupler lengths enable stronger Fano interference, resulting in steeper resonance slopes that are advantageous for high-resolution refractive index sensing.

**Figure 4:**
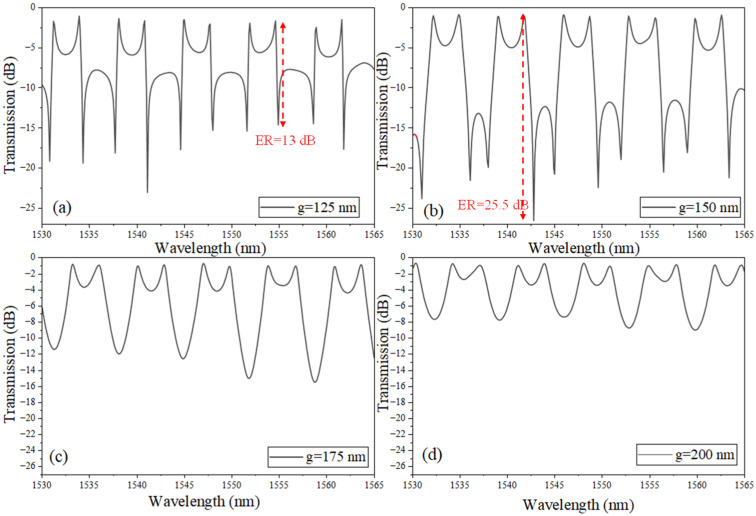
Transmission spectrum of the coupled-SLRs: (**a**) g = 125 nm, (**b**) g = 150 nm, (**c**) g = 175 nm, and (**d**) g = 200 nm. Note: R = 3 µm, L_DC_ = 2 µm, and L_f_ = 52.6 µm.

Figure 4 investigates how a uniform change in the coupling gap (g) across all three coupling regions (the two SLR directional couplers and the inter-SLR coupling section) modifies the transmission response of the coupled-SLR CROW. For the smallest gaps, g = 125 nm in Figure 4a and g = 150 nm in Figure 4b, the evanescent overlap is strong, enabling efficient loading of the resonant pathway and appreciable mixing with the broadband transmission channel. Under these conditions, the spectrum displays pronounced Fano resonances: asymmetric line profiles with steep slopes and high extinction (13 dB to 25.5 dB), including the characteristic double-dip structure arising from two interference solutions (two hybridized resonant conditions) within a single free-spectral interval. The asymmetry and depth here indicate that both the discrete (cavity) and continuum (bus/feedback) pathways contribute comparably, maximizing the interference contrast.

As the gap (g) increases to 175 nm (Figure 4c) and further to 200 nm (Figure 4d), the coupling weakens across all sections, which suppresses the discrete–continuum interference responsible for Fano lineshapes. Consequently, the spectral response transitions to more symmetric, weakly modulated features (eventually approaching near-flat transmission), with the earlier double-dip behavior disappearing. In this weak-coupling regime, only a small fraction of the field is coupled into the resonant loop, and inter-SLR mixing is minimal; the response is therefore dominated by the broadband path rather than by interference between comparable channels.

**Figure 5:**
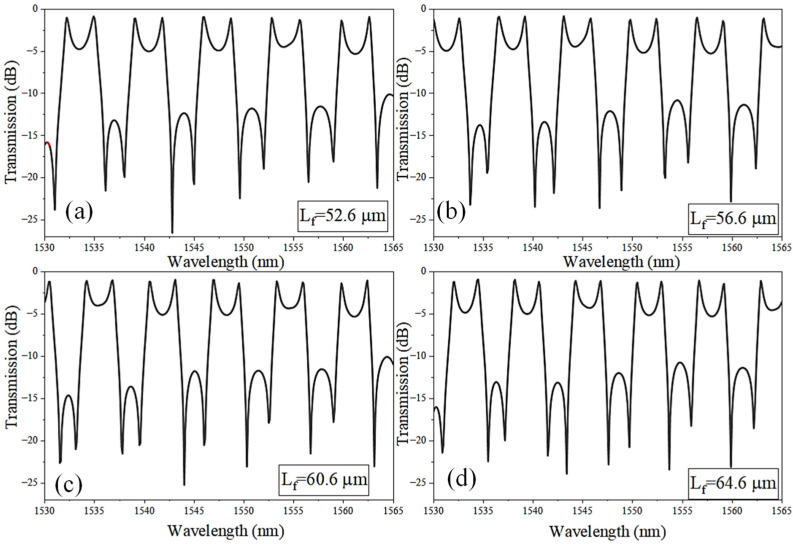
Transmission spectrum of the coupled-SLRs: (**a**) L_f_ = 52.6 µm, (**b**) L_f_ = 56.6 µm, (**c**) L_f_ = 60.6 µm, and (**d**) L_f_ = 64.6 µm. Note: R = 3 µm, L_DC_ = 2 µm, and g = 150 nm.

Figure 5 illustrates the influence of the feedback-waveguide length (L_f_) on the spectral response of the coupled-SLR CROW device. Because the Sagnac-loop radius, coupling gap, and directional-coupler length are held constant, variation in L_f_ becomes the dominant mechanism for modifying the overall round-trip optical length (L_rt_). An increase in L_f_ introduces additional phase accumulation in each circulation cycle, resulting in a systematic shift in the resonance positions. This behavior stems from the fact that the resonance condition is determined by the total accumulated round-trip phase, so modifying only the feedback-path length changes the phase balance between the discrete SLR-mediated resonant pathway and the broadband transmission channel.

A direct consequence of increasing L_f_ is a reduction in the device’s FSR. Because the FSR is inversely proportional to the round-trip optical length, Δλ ≈ λ^2^/(ngL_rt_), a longer feedback waveguide increases L_rt_ and causes adjacent resonances to move closer together. This trend is clearly evidenced in Figure 5a–d: as L_f_ increases from 52.6 µm to 64.6 µm, the resonances become progressively more closely packed, with the spacing between adjacent peaks shrinking from approximately 2.4 nm to 1.9 nm, reflecting the reduction in FSR associated with the increased round-trip optical path length. Importantly, although the resonance positions shift and the FSR contracts, the characteristic asymmetric (Fano-type) line shape is preserved throughout the sweep, demonstrating that Fano interference remains strong even as the round-trip phase is modulated. This phase sensitivity highlights the role of the feedback path as a tunable degree of freedom that enables fine spectral engineering without altering the SLR geometry or coupling interfaces. Overall, varying L_f_ provides a straightforward means of adjusting both the spectral period and the resonance bias of the coupled-SLR cavity. The ability to engineer the FSR by modifying only the feedback-path length is advantageous for applications requiring specific resonance spacing or wavelength targeting, while retaining steep asymmetric features ensures that high-resolution refractive index sensing performance is maintained.

**Figure 6:**
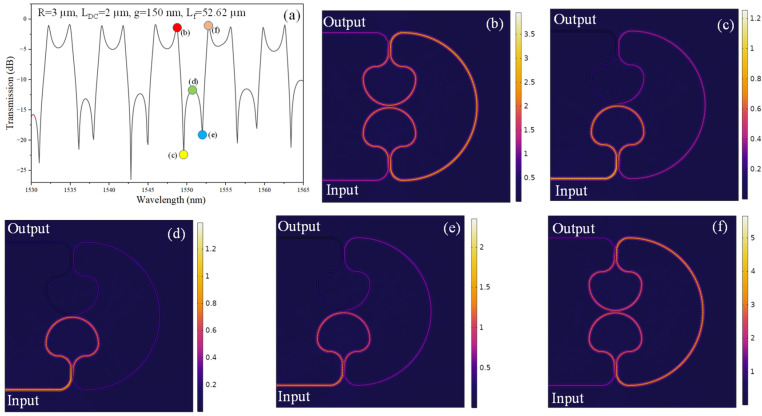
(**a**) Transmission spectrum of the coupled-SLRs. Norm. E-field distribution in the device for operational wavelength of (**b**) 1548.7 nm, (**c**) 1549.6 nm, (**d**) 1550.8 nm, (**e**) 1552 nm, and (**f**) 1552.8 nm.

Figure 6a correlates the device transmission spectrum with the spatial field evolution inside the coupled-SLR CROW at selected excitation wavelengths. The positions at which the field distributions are extracted are marked on the spectral curve and displayed in Figure 6b–f. At λ = 1548.7 nm, shown in Figure 6b, the device operates off-resonance, and the optical field predominantly follows the bus/feedback path, with only weak energy coupling into the SLR cavities. As the excitation wavelength approaches the asymmetric Fano feature, namely λ = 1549.6 nm, 1550.8 nm, and 1552.0 nm, corresponding to Figure 6c, 6d, and 6e, respectively, pronounced field localization is observed exclusively in SLR-1.

Across these three cases, negligible field buildup is seen in SLR-2, indicating that only a single resonant pathway contributes significantly to the scattering process. This asymmetric excitation condition, combined with the broadband transmission channel provided by the feedback waveguide, underpins the formation of the characteristic Fano lineshape. When the wavelength detunes further from resonance to λ = 1552.8 nm, as illustrated in Figure 6f, the optical field no longer remains confined within the SLR-1 and instead returns to the direct propagation path, resulting in high transmission. Collectively, these field maps verify that under the examined biasing conditions, the SLR-1 acts as the dominant resonant element, while SLR-2 plays only a minor role. The interaction between this single-cavity resonant channel and the continuum-like transmission through the bus/feedback path gives rise to the observed Fano interference and associated spectral asymmetry.

**Figure 7:**
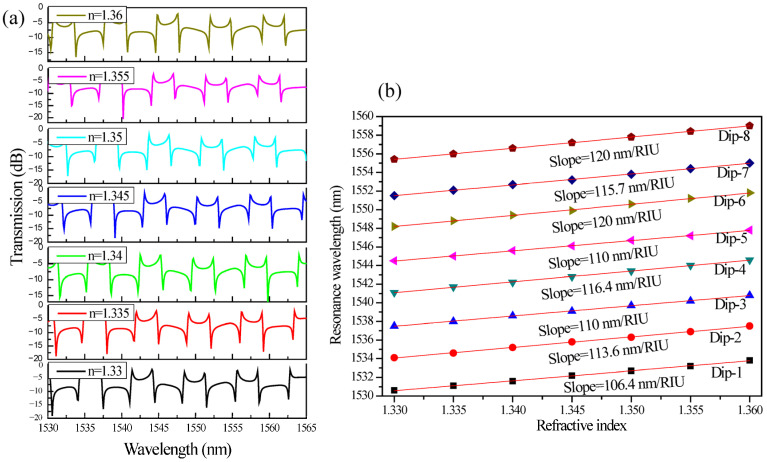
(**a**) Transmission spectrum of the coupled SLRs with ridge feedback waveguide for n = 1.33 to 1.36, (**b**) Resonance wavelength versus refractive index plot for Dip-1 to Dip-8. Note: R = 3 µm, L_DC_ = 2 µm, g = 150 nm, and L_f_ = 52.62 µm.

Figure 7a illustrates the transmission spectra of the coupled-SLR-based CROW sensor for refractive indices ranging from n = 1.33 to n = 1.36, while Figure 7b quantifies the corresponding resonance wavelength shifts for eight characteristic dips (Dip-1 → Dip-8). The refractive index range from 1.33 to 1.36 was chosen to reflect practical sensing conditions typically found in aqueous environments. Since water, with a refractive index of approximately 1.33 at 1550 nm, is commonly used as the reference medium, small variations within this range can reflect changes in solute concentration, biomolecular interactions, or chemical composition. Studying this narrow interval, therefore, allows the sensor performance to be evaluated under realistic conditions while remaining within the linear operating regime relevant to label-free detection. A clear and systematic red shift in the resonance minima is observed with increasing ambient refractive index, confirming that the optical mode exhibits strong evanescent-field interaction with the external analyte. The preservation of the distinct Fano-type asymmetric lineshape across the refractive index variation indicates stable interference between the discrete resonant channel, supported primarily by one SLR, and the broadband transmission channel through the feedback waveguide. This robustness to perturbations demonstrates that the coupled-SLR configuration maintains spectral integrity and high-Q resonance even when the surrounding refractive index changes, a key attribute for high-precision refractometric sensing.

Quantitatively, the linear fits in Figure 7b yield sensitivities (S = Δλ/Δn) of 106.4, 113.6, 110, 116.4, 110, 120, 115.7, and 120 nm/RIU for Dips 1 to 8, respectively. These values indicate a narrow variation range, demonstrating stable and reproducible phase modulation across multiple resonance orders. The slight fluctuation in sensitivity among different dips can be attributed to localized dispersion effects and variations in the effective confinement of the counter-propagating modes within the coupled loops. The nearly linear dependence of resonance wavelength on refractive index for all eight modes validates the device’s operation in the perturbative regime, where Δλ ∝ Δn. This linearity simplifies sensor calibration and ensures predictable performance over the operating range. Moreover, the comparable slopes among successive dips confirm uniform phase accumulation and negligible modal mismatch between the SLRs and the feedback path.

**Figure 8:**
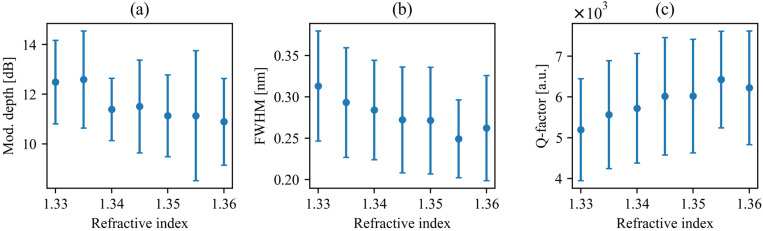
Average (dots) and standard deviation (vertical lines) values of modulation depth (**a**), FWHM (**b**), and Q-factor (**c**) plotted vs. refractive index for all considered resonances in the coupled SLRs with ridge feedback waveguide.

To further validate the sensing performance of the proposed system, we determined the changes in relevant parameters, i.e., modulation depth, FWHM, and Q-factor, as the refractive index of the surrounding medium increases. To better illustrate overall performance, we calculated the arithmetic average and standard deviation (SD) of those values for all observable resonances in the considered spectral range (Figure 8). The proposed system exhibits clear, monotonic, and gradual changes in its spectral characteristics with increasing refractive index of the surrounding medium, confirming its suitability for refractive index sensing. We emphasize that such moderate slopes are advantageous from an application perspective, as they indicate stable and predictable device operation. The absence of sharp fluctuations ensures that small refractive index perturbations do not induce unstable spectral behavior, which is essential for reliable sensing platforms.

Although the bars indicating standard deviation appear significant, they primarily originate from the limited spectral resolution of the simulations used to extract resonance parameters. In particular, the determination of FWHM, and consequently the Q-factor, is sensitive to discretization steps in spectral sampling. This numerical limitation increases the statistical spread of extracted values but does not affect the underlying physical trend. Importantly, all three parameters exhibit a clear, consistent monotonic change with increasing refractive index.

The gradual decrease in modulation depth suggests a weaker guiding of resonance wavelengths, while the simultaneous narrowing of the FWHM leads to a corresponding increase in the Q-factor. The reduction in FWHM indicated improved spectral selectivity, and the resulting increase in Q-factor confirms enhanced resonance sharpness. Overall, the observed trends, even in the presence of the apparent statistical spread, demonstrate a robust and consistent optical response, supporting the applicability of this structure for sensitive and reliable refractive index sensing.

## 4. Sensitivity Enhancement Mechanism

**Figure 9:**
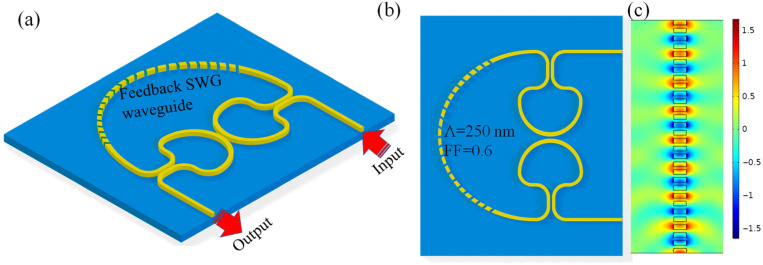
(**a**) 3D representation, (**b**) 2D representation, of CROW-based sensor employing SLRs with feedback SWG waveguide, (**c**) E_x_-field distribution in the SWG waveguide segment (Λ = 250 nm) at 1550 nm, illustrating the optimized configuration employed in the feedback waveguide. Note: Λ = SWG waveguide period and FF = fill factor.

Figure 9 illustrates the CROW sensor incorporating SLRs with a modified feedback waveguide comprising an SWG segment. In the 3D view (Figure 9a) and corresponding planar schematic (Figure 9b), the two SLRs remain interconnected through the feedback path, but the conventional strip-waveguide section is replaced with a periodic SWG structure. This engineered segment provides an additional degree of refractive index tunability through its enhanced modal interaction with the external medium while maintaining phase continuity within the coupled cavity.

The SWG section comprises periodically arranged silicon segments with pitch Λ = 250 nm. This subwavelength periodicity enables the guided mode to experience an effective refractive index lower than that of a solid strip waveguide, increasing the accessible evanescent field. As a result, changes in the analyte refractive index induce larger modal-index perturbations in the feedback branch, providing improved sensitivity without altering the SLR geometry or coupler interfaces. Figure 9c presents the simulated transverse electric-field distribution (E_x_) within the SWG feedback segment at 1550 nm. Strong field penetration into the cladding is observed, confirming that the periodic structure supports high-fraction evanescent interaction. By embedding the SWG only within the feedback path, the device simultaneously preserves the sharp asymmetric spectral response originating from the SLR-based coupled cavity and enhances refractive index sensitivity through engineered phase accumulation.

**Figure 10:**
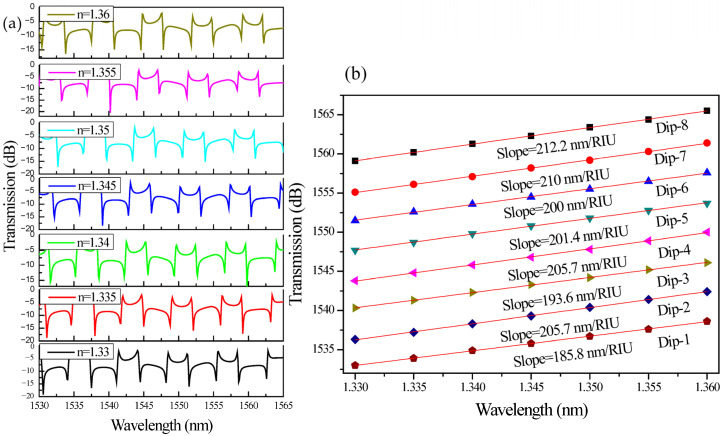
(**a**) Transmission spectrum of the coupled SLRs with SWG feedback waveguide for n = 1.33 to 1.36; (**b**) Resonance wavelength versus refractive index plot for Dip-1 to Dip-8. Note: R = 3 µm, L_DC_ = 2 µm, g = 150 nm, and L_f_ = 52.62 µm.

Figure 10a shows the simulated transmission spectra of the coupled-SLR CROW sensor employing an SWG feedback waveguide for refractive indices from n = 1.33 to 1.36. The SWG section enhances the evanescent-field interaction in the feedback branch by lowering the core’s effective index and increasing field penetration into the cladding. As the surrounding refractive index rises, the resonance dips exhibit pronounced red shifts, larger than those in the ridge feedback design, confirming improved modal sensitivity. The characteristic Fano-type asymmetry is retained across the sensing range, indicating that the periodic SWG does not disrupt the discrete–continuum interference but rather amplifies the refractive index-induced phase modulation within the cavity.

Figure 10b further quantifies this enhancement. The extracted sensitivities range from 185.8 to 212.2 nm/RIU, nearly twice those of the ridge feedback device (106–120 nm/RIU). This improvement arises from the increased effective-index contrast and higher overlap factor (Γ) provided by the SWG segment, which boosts the phase-modulation efficiency. The parallel slopes of the linear fits confirm uniform field confinement and consistent phase accumulation across all resonances. The linear dependence between refractive index change and spectral positions of dips demonstrates that the device remains within the perturbative regime, ensuring accurate and repeatable refractive index tracking. Overall, the SWG-assisted feedback configuration achieves substantial sensitivity enhancement through passive structural engineering while preserving the compact footprint and sharp Fano lineshape of the coupled-SLR cavity. This design nearly doubles the refractive index sensitivity, validating the SWG-integrated CROW as a promising platform for high-resolution, low-limit-of-detection (LOD) photonic sensing on SOI.

**Figure 11:**
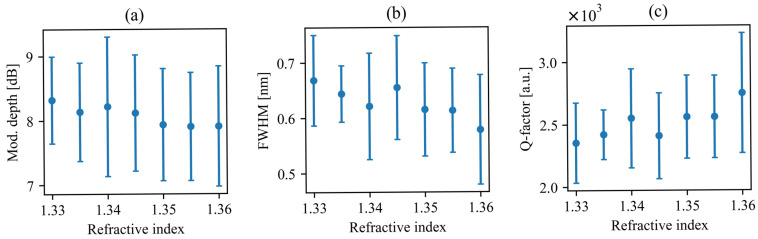
Average (dots) and standard deviation (vertical lines) values of modulation depth (**a**), FWHM (**b**), and Q-factor (**c**) plotted vs. refractive index for all considered resonances in the coupled SLRs with SWG feedback waveguide.

To fully investigate the sensing performance of the SWG-based configuration, we analyze the average and standard deviation of modulation depth, FWHM, and Q-factor of all resonances in the considered spectral range in Figure 11, following the same methodology as in the previous case.

Similar to Figure 8, the variations within the considered refractive index change are gradual. This moderate but systematic trend confirms stable operating conditions, in which resonance properties change predictably without abrupt changes. Despite an apparently large standard deviation, again mainly attributed to the finite spectral resolution of the simulations, the mean values exhibit a clear monotonic trend. This preserved tendency confirms the robustness of the sensing mechanism.

Compared to the ridge configuration, the SWG-based system exhibits a lower but more stable modulation depth across the investigated RI range, indicating improved retention of signal contrast under varying conditions. However, in contrast to the previous case, the FWHM values are higher, which results in correspondingly lower Q-factor and indicates broader resonances. While this leads to slightly reduced spectral sharpness, the configuration compensates through enhanced sensitivity (expressed in nm/RIU), enabling more pronounced resonance shifts in response to small variations in refractive index.

Overall, the comparison shows that the two configurations involve a trade-off between resonance sharpness (a higher Q-factor in the first design) and increased wavelength sensitivity (a higher nm/RIU in the SWG-based design). Despite the broader resonances, the monotonic and stable parameter evolution confirms reliable operation of the modified structure for refractive index sensing.

The LOD was estimated using a wavelength-interrogation approach consistent with practical measurements performed with an optical spectrum analyzer (OSA). In this method, a refractive index variation Δn induces a resonance shift Δλ = SΔn, where S = Δλ/Δn is the wavelength sensitivity of the sensor. The smallest detectable refractive index change is therefore limited by the minimum wavelength shift that can be resolved by the interrogation instrument. Assuming a typical OSA spectral resolution of 15 pm (0.015 nm) in the C-band, the LOD can be conservatively approximated as follows:

(5) $$LOD=\frac{∆\lambda_{OSA}}{S}$$

Using the simulated sensitivities of 106.4–120 nm/RIU for the coupled SLRs with ridge feedback waveguide configuration and 185.8–212.2 nm/RIU for the coupled SLRs with SWG feedback waveguide, the corresponding LOD is estimated to be approximately (1.25–1.41) × 10^−4^ RIU for the ridge feedback design and (7.07–8.07) × 10^−5^ RIU for the SWG feedback-assisted design. These results demonstrate a clear improvement in detection capability due to the incorporation of the SWG under realistic measurement conditions.

In practical wavelength-interrogation systems, the minimum detectable wavelength shift is influenced not only by the nominal spectral resolution of the OSA but also depends on the resonance linewidth, noise characteristics, and the specific interrogation and fitting procedure employed. A more realistic estimation can be obtained by relating the wavelength estimation uncertainty to the resonance full width at half maximum (FWHM) and the SNR, such that the minimum resolvable wavelength shift can be approximated as scaling with FWHM/SNR. This relation should be interpreted as an order-of-magnitude estimate, since the exact proportionality depends on the interrogation method (e.g., peak tracking, curve fitting, slope-based detection, or phase-sensitive measurement) and the noise model. For the ridge feedback configuration, with FWHM ≈ 0.28 nm and average sensitivity ≈ 110 nm/RIU, the resulting LOD varies depending on the effective SNR of the measured system. For typical laboratory SNR values between 10 and 20, the estimated LOD lies in the range of approximately 10^−4^ to a few ×10^−4^ RIU. Under more conservative low-SNR assumptions, the LOD may approach the ~10^−3^ RIU level. These considerations indicate that practical detection capability depends strongly on system noise and interrogation methodology. Furthermore, the steep spectral slopes associated with the Fano-type resonance enable alternative interrogation approaches, such as slope-based [27] or phase-sensitive detection [28], which can provide improved effective resolution compared to simple dip tracking with a broadband OSA.

To contextualize the performance of the proposed coupled-SLR CROW sensor, Table 2 summarizes recent refractive index sensors reported across different photonic platforms, including Si_3_N_4_, SOI, and hybrid plasmonic waveguides. The comparison highlights key performance metrics, including wavelength sensitivity, Q-factor, fabrication complexity, and LOD. Conventional microring and SWG-based resonators can achieve high sensitivity but often require complex geometries or careful parameter control during design and fabrication. In contrast, the coupled-SLR CROW devices introduced in this work offer competitive sensitivity with a structurally simple configuration and fewer coupled elements. This comparison highlights the ability of the proposed architecture to balance sensitivity with design simplicity and flexible spectral engineering.

## 5. Conclusions

A compact and highly sensitive refractive index sensor based on a coupled resonator optical waveguide (CROW) employing Sagnac loop reflectors (SLRs) on a silicon-on-insulator (SOI) platform has been designed and numerically analyzed. The coupled-SLR configuration, enhanced with a feedback loop, provides strong phase control and supports Fano-type asymmetric resonances with steep spectral slopes, thereby enabling precise detection of refractive index variations. A systematic investigation of structural parameters, including loop radius, coupling length, and feedback-path length, demonstrated their distinct influence on resonance asymmetry, free spectral range, and overall spectral performance.

The sensor achieves consistent and monotonic resonance wavelength shifts with sensitivities exceeding 100 nm/RIU and an average Quality factor above 5000 in the ridge feedback configuration. Incorporation of an SWG feedback segment further enhances evanescent-field interaction, achieving sensitivities up to ~212 nm/RIU and an average Quality factor exceeding 2500 without increasing the device footprint or complexity. The SWG-assisted CROW architecture, therefore, combines high sensitivity, tunability, and design flexibility within a simple, monolithic layout.

The proposed structure is fully compatible with standard SOI fabrication processes, including CMOS lithography and dry etching, and can be integrated with microfluidic channels to enable real-time measurements. The investigated refractive index range (1.33–1.36) corresponds to typical aqueous media, allowing detection of water-based chemical and biochemical analytes such as saline, glucose, or glycerol solutions, where small composition changes induce measurable refractive index variations.

These findings confirm the potential of coupled-SLR CROW devices as an efficient and scalable solution for high-resolution refractometric sensing. The demonstrated phase-engineering approach and Fano-resonance control can be further extended to multi-parameter biosensing, temperature-compensated detection, and integrated photonic systems that require compact, high-performance spectral filtering.

## Figures and Tables

**Table 1:** Geometric parameters of the device used in this study.

Parameter	Expression	Value
R	Radius of Sagnac loop	2 µm to 5 µm
g	The gap between the waveguides, and also, the gap between the two SLRs	125 nm to 200 nm
L_DC_	Length of the directional coupler	2 µm to 9 µm
W	Width of the waveguide	400 nm
L_f_	Length of the feedback waveguide	π × (R × 5 + LDC + g − W)
C-band	Operational wavelength range	1530 nm to 1565 nm

**Table 2:** Performance comparison of refractive index sensors based on various integrated photonic platforms and resonator architectures.

Material Platform	Device Structure	Sensitivity (nm/RIU)	Q-Factor	LOD (RIU)	Numerical/ Experimental	Fabrication Challenges	Ref.
Si_3_N_4_	Ring resonator with slot waveguide	70	750	-	Numerical	High	[29]
SOI	Hybrid dual slot SWG ring resonator	1005	2.2 × 10^4^	6.86 × 10^−5^	Numerical	High	[30]
SOI	SWG ring resonator	1012	1219	-	Numerical	High	[31]
SOI	Ring resonator	120	10^5^	10^−6^	Numerical	Low	[6]
Hybrid plasmonic	SWG Racetrack resonator	377.1 to 477.7	312.8 to 346.5	-	Numerical	High	[32]
Hybrid plasmonic	MZI	160	-	-	Experimental	High	[33]
Si_3_N_4_	Resonant cavity	201.5 to 341.5	3.9 × 10^4^	1.2 × 10^−4^ to 2 × 10^−4^	Experimental	Moderate	[14]
SOI	Coupled SLRs with ridge feedback waveguide	106.4 to 120	3835.8 to 7842.1	1.25 × 10^−4^ to 1.41 × 10^−4^	Numerical	Low	This work
SOI	Coupled SLRs with SWG feedback waveguide	185.8 to 212.2	1910.8 to 3790.2	7.07 × 10^−5^ to 8.07 × 10^−5^	Numerical	Moderate	This work

## Data Availability

The data supporting the findings in this work are available from the corresponding author upon reasonable request.
